# TREM1 is essential for maintaining stemness of liver cancer stem-like cells in hepatocellular carcinoma

**DOI:** 10.3389/fimmu.2025.1618342

**Published:** 2025-07-03

**Authors:** Arsha Sreekumar, Ashwin Ajith, Kenza Mamouni, Daniel D. Horuzsko, Anatolij Horuzsko

**Affiliations:** Georgia Cancer Center, Augusta University, Augusta, GA, United States

**Keywords:** TREM1, cancer stem cells, hepatocellular carcinoma, oncogenesis, chemotherapy

## Abstract

**Introduction:**

Hepatocellular carcinoma (HCC) is the most common primary liver cancer and a leading cause of cancer-related mortality worldwide. While the Triggering Receptor Expressed on Myeloid Cells 1 (TREM1) is well-known for its role in amplifying inflammation within the tumor microenvironment (TME), its tumor-intrinsic role remains poorly defined. Liver cancer stem-like cells (LCSLCs), charecerized by expression of CD133 and EpCAM, are critical for HCC initiation, metastasis, recurrence, and therapy resistance.

**Methods:**

We used flow cytometry to assess TREM1 expression in LCSLCs and employed CRISPR-Cas9 gene editing to knock out TREM1 in HCC cell lines. Functional assays, including proliferation, migration, apoptosis, clonogenicity, and spheroid formation, were performed. Cell line-derived xenograft (CDX) models were used to evaluate in vivo tumorigenicity. Transcriptomic profiling was conducted to explore downstream effects of TREM1 deletion. Additionally, a pharmacological inhibitor of TREM1 (VJDT) was used to validate the therapeutic potential of targeting TREM1 *in vivo*.

**Results:**

TREM1 was highly expressed in CD133^+^EpCAM^+^ LCSLCs. Knockout of TREM1 significantly impaired proliferation and migration while promoting apoptosis in HCC cells. In LCSLCs, TREM1 silencing reduced clonogenic ability and spheroid formation, indicating loss of self-renewal and stemness. In CDX models, TREM1-deficient LCSLCs exhibited markedly reduced tumorigenicity. Transcriptomic analysis revealed distinct, context-dependent gene expression changes in nuclear and extracellular signaling pathways following TREM1 loss. Pharmacologic inhibition of TREM1 with VJDT recapitulated the tumor-suppressive effects observed in genetic models.

**Discussion:**

Our findings establish TREM1 as a critical tumor-intrinsic regulator of LCSLC survival and tumorigenic potential, independent of its known immunomodulatory role in the TME. Targeting TREM1 may therefore represent a promising dual-action therapeutic strategy to disrupt both cancer stem-like cell function and the pro-inflammatory tumor milieu in HCC.

## Introduction

Liver cancer is the sixth most frequently diagnosed cancer and the fourth leading cause of cancer-related deaths worldwide (1, 2). Hepatocellular carcinoma (HCC), the most common type of liver cancer, is strongly associated with chronic liver diseases, including cirrhosis, hepatitis B or C infection, and prolonged alcohol consumption (3, 4). Despite remarkable progress in surgical techniques and chemo/immunotherapies, long-term survival rates after surgical resection or locoregional therapies remain low (5, 6). This underscores the imperative need to delineate the underlying molecular mechanisms in HCC oncogenesis and progression.

Triggering Receptors Expressed on Myeloid Cells (TREM1), a proinflammatory molecule within the immunoglobulin superfamily, plays a crucial role in the development and progression of HCC (7). TREM1 intensifies hepatic inflammation by promoting the secretion of proinflammatory mediators, fostering fibrosis and tumorigenesis in HCC (8). While our lab has previously established the proinflammatory role of TREM1 in the tumor microenvironment (TME), its direct involvement in the cancer cells themselves, independent of its TME effects, has yet to be explored (9–11).

To address this gap, we investigated the role of TREM1 in two well-established liver cancer cell lines Huh7 and HepG2 using control and CRISPR-Cas9 *TREM1* KO cells. Initial studies revealed that TREM1 silencing inhibited cell proliferation, disrupted the cell cycle, and promoted apoptosis in both cell lines. Interestingly, preliminary flow cytometry analysis demonstrated that TREM1 silencing significantly reduced the proportion of CD133^+^EpCAM^+^ liver cancer stem-like cells (LCSLCs). LCSLCs are a small subset of tumor cells with high self-renewal capacity, strong tumor initiation potential, and unlimited differentiation ability (12, 13). These cells are also believed to be resistant to conventional chemotherapy or radiotherapy, contributing to tumor recurrence and metastasis through epithelial-mesenchymal transition (EMT) (14–16).

Various well-established cell surface markers, including CD13, CD24, CD44, CD133, and EpCAM, have been identified to characterize LCSLCs (17). However, liver tumors are highly heterogeneous, and this heterogeneity is amplified by the ability of LCSLCs to differentiate into diverse cell populations. Therefore, using any single marker or combination of markers may only identify a small subset of cancer stem-like cells. For our study, we selected CD133 and EpCAM as LCSLC markers, as they are well-established in both Huh7 and HepG2 cell lines (12, 18). CD133^+^ liver cancer cells are known for their enhanced tumorigenic potential, resistance to chemotherapy, and higher self-renewal capacity. Furthermore, expression of CD133 is correlated with poor prognosis and aggressive tumor behavior (19–21). Similarly, EpCAM^+^ cells in HCC exhibit high tumorigenicity and resistance to apoptosis (18, 22). The co-expression of CD133 and EpCAM in LCSLCs marks a subpopulation with significant stem cell-like properties, contributing to tumor growth and recurrence (12, 23). These cells are thought to drive major challenges in liver cancer treatment, such as early recurrence, metastasis, and angiogenesis (24, 25). Given their critical role, isolating and therapeutically targeting these cells are paramount to obtaining long-term overall survival.

In this study, we utilized a combination of CRISPR-Cas9 genome ablation of TREM1 and its pharmacological inhibition by a novel small molecule inhibitor, VJDT, to characterize the impact of TREM1 silencing in cancer cells directly (9). Our findings suggest that targeting TREM1 in Huh7 and HepG2 cell lines reduces the proportion of CD133^+^EpCAM^+^ LCSLCs. We hypothesize that TREM1 plays a critical role in maintaining the stemness of this subpopulation of LCSLCs and its inhibition can specifically target these cells to restrict tumor proliferation. To investigate this further, we isolated these cells using Fluorescence-Activated Cell Sorting (FACS) and Magnetic-Activated Cell Sorting (MACS) and performed a series of *in vitro* assays, including cell cycle and apoptosis assays. Additionally, we employed cell line-derived xenograft (CDX) models and bulk RNA-sequencing to determine the role of TREM1 in CD133^+^EpCAM^+^ LCSLCs in liver cancer. This study is more focused on addressing the intrinsic role of TREM1 in cancer cells, separate from its well-established effects in TME.

## Materials and methods

### Cell lines and cell culture

Custom-developed CRISPR-Cas9 Huh7 control (YC-D001, Ubigene), Huh7 *TREM1* KO (YKO-H838, Ubigene), HepG2 control (YC-C001, Ubigene), HepG2 *TREM1* KO (YKO-H838, Ubigene) and Hep3B (HB-8064, ATCC) cell lines were used. The cells were cultured in Dulbecco’s Modified Eagle’s Medium (DMEM, MT10013CV, Corning) supplemented with 10% fetal bovine serum (FBS, SH3039602, Cytvia) and 100 u/mL penicillin/streptomycin (15-140-163, Gibco) in a 37°C incubator under humidified 5% CO2. For primary culture of human liver tumor cells, tissues were processed under sterile conditions by mincing into small fragments (~1–2 mm³) using sterile scalpels. The fragments were enzymatically digested in a solution of collagenase IV (17-104-019, Gibco™) and DNase I (AAJ62229MB, Thermo Scientific) at 37°C for 60 minutes with gentle agitation. Following digestion, the cell suspension was passed through a 70 µm cell strainer to remove undigested debris. The filtrate was centrifuged at 400 × g for 5 minutes, and the resulting cell pellet was washed with PBS. Cells were resuspended in DMEM/F12 media (SH3002301, Cytiva HyClone™) supplemented with 10% FBS, 20 ng/mL epidermal growth factor (354010, Corning), 5 µg/mL insulin (12-585-014, Gibco™), 1 µg/mL hydrocortisone (AC352450010, Thermo Scientific), and 100 U/mL penicillin/streptomycin, and then plated onto collagen-coated tissue culture dishes. Cultures were maintained at 37°C in a humidified incubator with 5% CO_2_. Medium was replaced every 2–3 days, and cells were passaged when they reached 70–80% confluence.

### Generation and validation of TREM1 knockout cell lines

TREM1 knockout (TREM1-KO) Huh7 and HepG2 cell lines were generated and validated by Ubigene (Guangzhou, China) using their proprietary Red Cotton™ CRISPR-Cas9 gene editing platform. Exon 2 of the human TREM1 gene was targeted using the following sgRNAs: for Huh7, sgRNA1: GTAGTCACATTTCACATCCAGGG and sgRNA2: AGCATGTGAGGCTCCTTGGGAGG; for HepG2, sgRNA3: ACTGGATGGGAATTCTTTGAAGG and sgRNA4: GAAAGCTTGGCAGATAATAAGGG. Electroporation parameters were optimized for each cell line (Huh7: 1600 V, 10 ms, 3 pulses; HepG2: 1230 V, 10 ms, 2 pulses).Single-cell clones were isolated by limiting dilution and screened for deletions in exon 2 by PCR and Sanger sequencing. RT-PCR was performed to confirm the loss of TREM1 transcript expression. All gene editing and validation steps were conducted by Ubigene, and fully characterized knockout clones were provided for downstream experiments. Representative validation data, including PCR and sequencing results for both HepG2 and Huh7 clones are shown in **Supplementary Figures 1 and 2**.

### Animal studies

NOD scid γ (NOD.Cg-Prkdc^scid^Il2rgtm1Wjl/SzJ, JAX 005557) mice aged 4–6 weeks were purchased from Jackson Laboratory. Age- and sex-matched animals were included in all experiments. The mice were housed in a climate-controlled specific pathogen-free environment within the Augusta University animal facilities. The study protocol (2008-0051) was approved by Augusta University Institutional Animal Care and Use Committee (IACUC).

### Human tumor samples

HCC patient samples were collected from Georgia Cancer Center Biorepository Bank following protocols approved by Institutional Review Board Committee. Informed consent was obtained from all subjects providing fresh tissues. The samples were snap frozen in liquid nitrogen. All experiments were carried out in accordance with relevant guidelines and regulations.

### Data analysis

HCC and normal tissue expression profiles were obtained from The Cancer Genome Atlas (TCGA) database, in which mRNA expression level of TREM1 was analyzed. The expression data was also cross-referenced with TCGA clinical data. The Kaplan–Meier estimator was used to produce survival curves and assess the prognostic significance of TREM1 in HCC. Patients with aggressive HCC were categorized into high and low TREM1 expression groups based on 50% quantile as cut off for TREM1 expression.

### Western blot analysis

Cells were cultured in 6-well plates until they reached 80% confluency. Cells were lysed in 40µl lysis buffer (FNN0021, Invitrogen) supplemented with 1mM PMSF and protease inhibitor cocktail (A32955, Thermo Fisher Scientific). 80µg of protein were resolved and separated by 4-20% Mini-PROTEAN^®^ TGX™ Precast gels (45461094, BIO-RAD), transferred onto PVDF membranes (IPFL85R, Millipore). Followed by blocking with SuperBlock blocking buffer (PI37580, Thermo Fisher Scientific) for 1h, membranes were incubated with primary antibodies - TREM1 monoclonal (FERMA548755, Invitrogen,1:1000), CD133 Rabbit polyclonal (A0219, Abclonal, 1:1000), Epcam Rabbit polyclonal (A23654, Abclonal, 1:1000), OCT4 Rabbit polyclonal (A7920, Abclonal, 1:1000), Nanog Rabbit polyclonal (A3232, Abclonal, 1:1000), Sox2 Rabbit polyclonal (A19118, Abclonal, 1:1000), β Actin (MAB8929SP, R&D Systems,1:1000), α tubulin (sc-53029, Santa Cruz Biotechnology, 1:1000) at 4°C overnight. After washing in TBST, the membranes were incubated with secondary antibodies - goat anti-mouse HRP-conjugated secondary antibody (AP127PMI, Millipore Sigma, 1:10,000), HRP-conjugated Anti-Mouse IgG (MAB8929-SP, R&D Systems, 1:10,000) and HRP-conjugated anti-Rabbit (AS014, Abclonal, 1:10,000) for 1h at room temperature (RT). After washing, the protein signals were visualized using ECL detection (45-002-401, Cytvia Amersham).

### Quantitative RT-PCR assay

Total RNA was isolated using RNeasy Mini kit (74104, Qiagen). The cDNA was synthesized using RT^2^ First Strand Kit (330404, Qiagen). qRT-PCR was performed using RT^2^ SYBR Green qPCR Mastermix (330502, Qiagen) with an ABI Step OnePlus Real-Time PCR system (Applied Biosystems) according to the operator’s manual. The gene expression values were normalized to GAPDH. Each experiment was carried out in triplicate. Primers are provided in **Supplementary Table**.

### Immunohistochemistry

HCC tumor and normal tissues (NCT046) were obtained from US Biomax. The sections were deparaffinized for multiplexed staining using the Opal protocol. According to the manufacturer’s instructions, paraffin-embedded tumor tissue samples were heated at 57°C overnight. Residual paraffin was removed using xylene, and the tissues were rehydrated through a series of graded alcohols to distilled water. Antigen retrieval was performed by microwave heating in AR6 solution (2620012, PerkinElmer). After cooling and washing with TBST, the tissues were blocked with antibody diluent (ARD1001EA, PerkinElmer) for 30 minutes at RT. The tissue sections were then incubated with primary antibody for 1 hour. After washing with TBST, the slides were incubated with HRP-conjugated secondary antibody (ARH1001EA, PerkinElmer) for 30 minutes at RT, followed by staining with Opal 650 (FP1496A, PerkinElmer) diluted at a 1:100 ratio in 1X amplification diluent (FP1498, PerkinElmer) for 10 minutes at RT. This process was repeated for staining with both primary antibodies: TREM1 Rabbit polyclonal antibody (11791-1-AP, Proteintech, 1:200) and alpha-Fetoprotein monoclonal antibody (13-949-982, Invitrogen, clone: 1E8, 20 µg/mL). Afterward, the sections were washed with TBST, and the nuclei were stained with ProLong™ Gold antifade reagent with DAPI (Invitrogen, P36931). Multiplexed imaging was conducted using the Opal 7-Color Manual IHC Kit, and the multiplexed sections were imaged at x20 magnification on the Vectra Automated Quantitative Pathology Imaging System.

### Flow cytometry

Huh7 and HepG2 control and CRISPR-Cas9–mediated TREM1 KO cell lines were seeded in 6-well plates and cultured under standard culture conditions (see Cell Lines and Cell Culture). Once the cultures reached approximately 80% confluency, 10^6^cells were harvested and suspended in ice-cold FACS buffer (00422226,eBioscience) and incubated with following antibodies (all from BioLegend) at 4°C for 45 minutes in dark: TruStain FcX (clone: 93, 101319, 1:50 dilution), FITC anti-human CD133 (397907, clone: W6B3C1, 5 µL in 100 µL staining volume) and APC anti-human EpCAM (369809, clone: CO17-1A, 5 µL in 100 µL staining volume). Cells were acquired on the Attune NxT Acoustic Focusing flow cytometry platform (Thermo Fisher Scientific) and data were analyzed on FlowJo v10.0. Forward versus side scatter (FSC versus SSC) gating was used to exclude dead cells. Patient samples, vehicle and VJDT treated tumors: Tumors were processed into a single-cell suspension using gentle MACS Octo Dissociater with Heaters (Miltenyi Biotech) in combination with the tumor dissociation kit (Miltenyi Biotech). 1 million cells were collected, incubated with antibodies and acquired as described above.

### Magnetic activated cell sorting

CD133^+^ cells were isolated using the CD133 Microbead Kit (130-097-049, Miltenyi Biotec). Briefly, cells were incubated with CD133 microbeads and then passed through an LS column (130-042-401, Miltenyi Biotec) placed in the magnetic field of a MACS separator (130-042-302, Miltenyi Biotec) attached to a MACS multistand (130-042-303, Miltenyi Biotec). The magnetically labeled CD133^+^ cells were retained in the column, while the unlabeled cells flowed through and were collected as the CD133^-^ fraction. After removing the column from magnetic field, the retained CD133^+^ cells were eluted and subsequently incubated with EpCAM microbeads (130-061-101, Miltenyi Biotec). These cells were loaded onto a magnetic column to collect CD133^+^EpCAM^+^ cells. The CD133^-^EpCAM^-^ cells were collected separately. Both fractions were subsequently cultured to obtain sufficient cells for further experiments.

### Fluorescence activated cell sorting

Cells were harvested using trypsin-EDTA to ensure a single-cell suspension. Cells were washed by centrifugation at 1500 rpm for 5 minutes at 4°C and resuspended in ice-cold FACS buffer. Cell viability was confirmed to be above 90%. Fc block was performed by adding 100 µL of Fc block diluted in FACS buffer (1:50 ratio) to each sample, followed by incubation on ice for 20 minutes. Cells were stained with FITC anti-CD133 and APC anti-EpCAM antibodies diluted in FACS buffer at 4°C for 45 minutes in dark. Cells were washed three times by centrifugation at 1500 rpm for 5 minutes and resuspension in ice-cold FACS buffer. Cells were sorted using Invitrogen™ Bigfoot™ Cell Sorter (Thermo Fisher Scientific). Cells were sorted based on CD133 and EpCAM expression. Sorted cells were collected into appropriate collection tubes, and the purity of sorted populations was analyzed, aiming for at least 90% purity. Unstained, single-stained, and isotype controls were included for accurate gating.

### CCK-8 assay

Cell Counting Kit-8 (CCK-8, GK10001, Glpbio) was used to examine cell proliferation. 1 ×10^4^ cells were cultured in 96 well plate for 0,24,48 and 72h. after incubating with 10µl CCK-8 solution at 37°C for 1hr, absorbance at 440nm was measured using microplate reader (biotek synergy htx reader). The experiment was conducted in triplicate.

### Transwell migration assay

Migration assay was conducted with transwell plate (07-200-174, Corning). Briefly, 1ml medium containing FBS was added in the lower chamber and 600µl media containing 6 ×10^4^ cells were added in the upper chamber. After incubating for 24hr, the transwell inserts were fixed in 4% paraformaldehyde for 10mins. Subsequently, the cells were stained with 0.05% crystal violet dissolved in PBS for 30 minutes. The transwell plates were then washed with PBS and the cells in the upper chamber were removed using cotton swabs. The cells that had migrated to the lower chamber were observed and counted under a microscope. The experiment was conducted in triplicate.

### Apoptosis assay using flow cytometry

Cells were collected, washed twice with cell staining buffer (420201), and resuspended in Annexin V Binding Buffer (422201) at a concentration of 1x10^6^ cells/mL. 100 µL of cell suspension was transferred into 5 mL test tube. 5µl each of FITC Annexin V (640905) and propidium iodide (421301) were then added to the cell suspension, followed by gentle vortexing. The cells were incubated at RT in the dark for 15 minutes. Subsequently, an additional Annexin V Binding Buffer was added, and the cells were immediately acquired on the Attune NxT Acoustic Focusing flow cytometry platform (Thermo Fisher Scientific) and data were analyzed on FlowJo v10.0. All reagents used were obtained from BioLegend.

### Apoptosis gene real-time PCR array

Total RNA was isolated using RNeasy Mini kit (74104, Qiagen). The cDNA was synthesized using RT2 First Strand Kit (330404, Qiagen). The first strand cDNA was mixed with RT2 SYBR Green qPCR Mastermix (330502, Qiagen), and aliquoted into the 96-Well RT2 Profiler PCR Array for apoptosis (PAHS-012A, SA Biosciences). This assayed 84 key genes associated with apoptosis. RT-PCR was performed using ABI Step OnePlus Real-Time PCR system (Applied Biosystems) according to the operator’s manual. GAPDH was used for data normalization.

### Cell cycle assay

Cells were cultured in 6-well plates until they reached 80% confluency. Cells were collected and pelleted. 5ml cold 70% ethanol was added dropwise to the pellet and incubated at -20°C for 2 hours. Cells were washed twice in cell staining buffer and resuspended. 100µl cell suspension containing 10^6^ cells were transferred into sample tubes and incubated with 20µl Alexa Fluor^®^ 488 anti-human Ki-67 Antibody (350507, BioLegend, clone: Ki-67) at RT for 30 minutes in the dark. After washing with cell staining buffer, a 0.5ml staining buffer and 10 µl PI solution were added to the sample tubes. Following this the cells were acquired on the Attune NxT Acoustic Focusing flow cytometry platform (Thermo Fisher Scientific) and data were analyzed on FlowJo v10.0.

### Clonogenicity assay

Cells were seeded in triplicate at a density of 500 cells/well in 6-well plates. After 14 days, the medium was aspirated, and cell colonies were washed twice with PBS. They were then fixed in 4% paraformaldehyde for 5 mins followed by staining with 0.05% crystal violet dissolved in PBS for 30mins. Excess dye was removed by rinsing twice with PBS. After drying, the colonies were counted under a microscope. The experiment was conducted in triplicate.

### Spheroid formation assay

5000 CD133^+^EpCAM^+^ control and *TREM1* KO cells per well were seeded on low attachment 6-well plates in serum free DMEM/F12 medium supplemented with 2% B27(12687-010, Gibco), 1% penicillin-streptomycin, 5 ng/mL epidermal growth factor (354010, Corning) and 10 ng/mL basic fibroblast growth factor (13256-029, Invitrogen). After 7 days of culture, spheres were counted and photographed under Keyence microscope. For pharmacological inhibition of TREM1, spheroids were treated with 10µM VJDT on day 7 and were counted on day 10. Both experiments were conducted in triplicate.

### *In vivo* tumorigenicity assay

For CDX model, 200µl cell suspension containing 5000 freshly sorted CD133^+^EpCAM^+^ control and *TREM1* KO cells mixed with Matrigel (356230, Corning) in a 1:1 ratio and were subcutaneously injected into the left flank of 5 weeks old NSG mice (n=6 per group). The mice were observed every other day for tumor growth for 60 days. After this, mice were sacrificed, and all tumor tissues were harvested for morphological assessment. For experiments involving pharmacological inhibition of TREM1, 1x10^6^ Huh7 cells were subcutaneously implanted onto 10 NSG mice. Mice were treated with either 20 mg/kg VJDT or vehicle (DMSO) intraperitoneally on day 10 after tumor cell injection and continued alternate days until day 22 (n=5 per group). VJDT is a novel TREM1 small molecule inhibitor that effectively blocks TREM1 signaling. VJDT design, development, dose optimization and toxicity studies are described in our prior work and is covered under the international patent application WO2022061226A1 (9). Tumor growth was measured alternate days using a digital caliper; tumor volumes were calculated using the formula V=L × W2 × (π/6), where L and W denote length and width of the tumor.

### Bulk RNA sequencing and analysis

CD133^+^EpCAM^+^ control and *TREM1* KO cells were sorted using FACS. Total RNA was isolated using Trizol reagent (5–596-026, Thermo Fisher Scientific) and purified using RNEasy mini kit (74104, Qiagen). The samples were submitted to Integrated Genomics Core Shared Resources at Augusta University (RRID: SCR_026483). Briefly RNA sequencing was carried out using an Illumina NovaSeq 6000, and filtered clean reads were mapped to a reference genome using hierarchical indexing for spliced alignment of transcripts 2 (HISAT2). Visualization of alignment results were verified using Integrative Genomics Viewer. RNA-seq data were analyzed using iDEP (integrated Differential Expression and Pathway analysis) (26). Differential expression analysis was performed using DESeq with Fold Change ≥1 and FDR < 0.05 set as screening criteria. Kyoto Encyclopedia of Genes and Genomes (KEGG) enrichment analysis was used to identify the significantly affected pathways during *TREM1* KO. Gene Ontology enrichment analysis for molecular function, biological process and cellular component was performed using IDEP.

### Statistics

Statistical analysis was conducted using GraphPad Prism 9 (version 9.0.0). Data are generally presented as mean ± SD, unless specified otherwise. Graphs display either group mean values ± SD for *in vitro* experiments or ± SEM for *in vivo* experiments, or individual values. If the data sets followed a normal distribution and comparisons were made between two experimental groups, an unpaired, two-tailed Student’s t-test was utilized. For *in vitro* studies, unpaired t-tests were used for comparing two groups, while for *in vivo* studies, a two-way ANOVA was employed for multiple comparisons of longitudinal tumor growth across various groups. A p-value of less than 0.05 was considered statistically significant.

## Results

### TREM1 is strongly expressed in liver hepatocellular carcinoma

**Figure 1:**
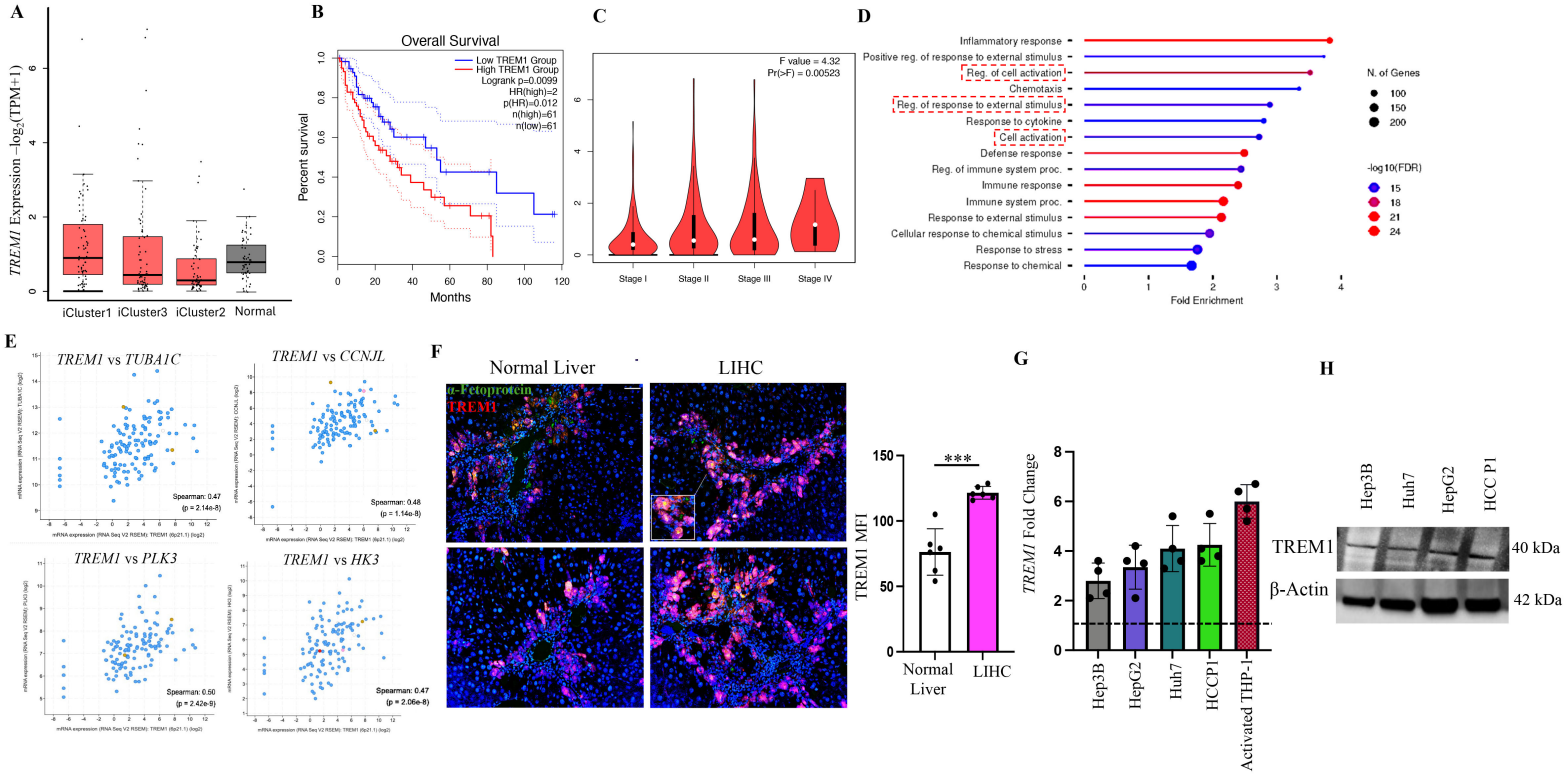
TREM1 is actively expressed in liver hepatocellular carcinoma. **(A)** Box plot shows higher TREM1 expression in iCluster1 and iCluster3 compared to iCluster2 and normal tissue (plotted using Graphpad Prism 9). **(B)** Kaplan-Meier survival curve shows significant correlation between TREM1 expression and worse overall survival in iCluster1 and 3 cohorts. **(C)** Violin plot shows correlation between TREM1 expression and tumor stages. Stage 4 tumor shows highest TREM1 expression. **(D)** Pathway analysis of top 200 TREM1-correlated genes (Spearman correlation>0.5) from iCluster 1 and 3. **(E)** *TUBA1C, CCNJL, PLK3*, and *HK3* expression associated with cell division exhibit positive correlation to TREM1 expression. **(F)** Immunofluorescence staining of LIHC patient samples shows significant overlap of TREM1 and α-feto protein. Original magnification, ×10; scale bar: 100μm. MFI = mean fluorescence intensity (n=6 per group) **(G)** RT-PCR confirms *TREM1* expression in HCC cell lines and HCC patient P1. TREM1 expression was analyzed as fold change with resting THP-1 used as baseline indicated by a dashed line (n=4 per group). **(H)** Western blot analysis detects TREM1 protein level expression at varying levels across all HCC cell lines tested. ***p<0.001.

To gain a comprehensive understanding of the role of TREM1 in liver hepatocellular carcinoma (LIHC), we analyzed the LIHC cohort from the TCGA database (27). The cohort was subdivided into three iClusters based on well-established parameters, including specific mutations (*TP53, CTNNB1*) and multi-omics data (27, 28). iCluster1 and iCluster3 represent patients with the more aggressive, rapidly proliferating LIHC and who have a worse prognosis, while iCluster2 patients exhibit more differentiated tumors and have better overall survival (27, 29). Interestingly TREM1 expression was consistently higher in iCluster1 and iCluster3 compared to iCluster2 and matched normal tissue (**Figure 1A**). Higher TREM1 expression also significantly correlated with worse overall survival in the combined iCluster1 and iCluster3 cohorts (**Figure 1B**, p<0.05). These data suggest that high TREM1 expression is associated with more aggressive tumors and poor prognosis for HCC patients. Additionally, TREM1 expression increased with tumor stage, with stage 4 showing the highest expression (**Figure 1C**). Pathway analysis of the top 200 TREM1-correlated genes (Spearman correlation > 0.5) revealed that the inflammatory response was the most enriched pathway (**Figure 1D**). Other pathways of interest included regulation of cell activation, response to external stimuli, and cell activation, which differ from the conventional inflammatory pathways associated with TREM1. Genes associated with cell division, such as *TUBA1C(*Tubulin Alpha 1c), *CCNJL(Cyclin J-Like)*, *PLK3(Polo-Like Kinase 3)*, and *HK3* (*Hexokinase 3)* (30–32), exhibited positive correlation with TREM1 expression and are associated with aggressive LIHC tumors (**Figure 1E**). To confirm our observations from the TCGA database, we performed immunofluorescence staining of LIHC tumor sections. This staining exhibited marked overlap between TREM1 and α-fetoprotein, a classical marker for HCC cells (33–35) (**Figure 1F**), indicating that the tumor cells themselves are positive for TREM1. The data also showed that TREM1 expression is significantly higher in LIHC sections compared to matched normal tissues (p<0.001). Additionally, analysis of Human Protein Atlas database revealed a moderate to high TREM1 protein expression in the cytoplasm/membrane of HCC sections (**Supplementary Figure 3**). To further validate our findings and explore the role of TREM1 in liver cancer at the cellular level, we assessed TREM1 expression in human HCC cell lines (Huh7, HepG2, Hep3b) and one primary HCC patient (P1). RT-PCR analysis revealed elevated expression of TREM1 across all groups compared to resting THP-1 cells, which have negligible TREM1 expression (36) (**Figure 1G**). Western blot analysis detected TREM1 protein levels across all HCC cell lines tested at varying levels (**Figure 1H**). Our data indicates that TREM1 is expressed in liver cancer cells and tissues. It also suggests that TREM1 has a more direct role within these cancer cells, separate from its traditionally recognized involvement in inflammation.

### TREM1 knockout suppresses proliferation and migration while inducing apoptosis in HCC

**Figure 2:**
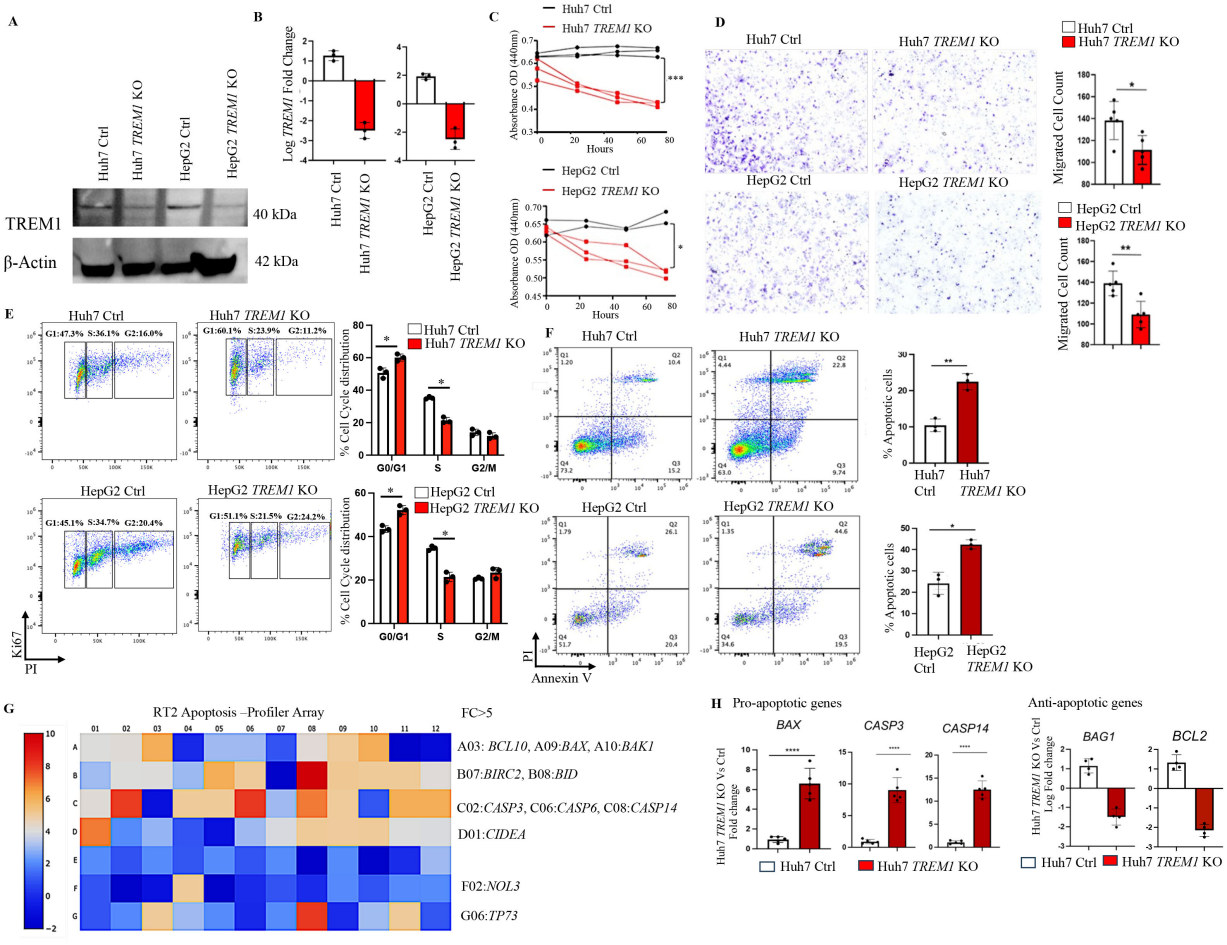
TREM1 knockout suppresses proliferation and migration while inducing apoptosis in HCC. **(A)** Western blot and **(B)** RT-PCR analysis confirms TREM1 knockout using CRISPR-Cas9 in Huh7 and HepG2 cell lines (n=3 per group). **(C)** Line graph shows CCK-8 assay assessing cell proliferation in control and *TREM1* KO Huh7 and HepG2 cell lines (n=2–3 per group). **(D)** Cell migration in Huh7 and HepG2 cell lines, both control and *TREM1* knockout groups assessed by *in vitro* transwell assay. Representative images of crystal violet staining captured at 24h. Number of migrated cells on each six-well plate counted in 3 independent experiments (n=3 per group) **(E)** Flow cytometry histogram plots depict Ki67-PI cell cycle analysis of control and *TREM1* KO Huh7 and HepG2 cell lines. Representative plot from 3 independent experiments performed in triplicate (n=3 per group). **(F)** Flow cytometry analysis using Annexin V-PI staining shows significant increase in apoptosis during TREM1 silencing in Huh7 and HepG2 cell lines (n=3 per group). **(G)** RT2 PCR Array analysis of human apoptotic gene expression. Heatmap shows the expression of 84 key genes associated with apoptosis. Upregulated genes (red) and downregulated genes (blue) in Huh7 *TREM1* KO cells shown. **(H)** Upregulation of pro-apoptotic genes and downregulation of anti-apoptotic genes in Huh7 *TREM1* KO cells compared to the control group plotted using GraphPad Prism (version 9) (n=4–5 per group). ****p<0.0001.

To determine the specific role of TREM1 in HCC, we employed the CRISPR-Cas9 system to knock out TREM1 expression in HepG2 and Huh7 cell lines. Western blot analysis confirmed a marked reduction in TREM1 expression in the KO cells, although faint TREM1 bands were still observed, which may represent residual truncated protein isoforms with negligible functional capacity (**Figure 2A**). To further validate the knockout efficiency, we performed RT-PCR (**Figure 2B**) and flow cytometry analysis (**Supplementary Figure 4**).Functional assays revealed that TREM1 silencing significantly inhibited cell proliferation (p<0.01), as demonstrated by CCK-8 assays, and reduced migration capacity in transwell assays in both Huh7 (p<0.05) and HepG2 (p<0.01) cell lines (**Figures 2C, D**). Additionally, Ki-67-based cell cycle analysis indicated a substantial decrease in the proportion of S-phase cycling cells (p<0.05) and a G1 cell cycle (p<0.05) arrest in Huh7 and HepG2 *TREM1* KO lines, suggesting impaired cell cycle progression (**Figure 2E**). These results highlight the critical role of TREM1 in promoting cancer cell proliferation. To assess the impact of TREM1 knockout on apoptosis, we analyzed the proportions of apoptotic cell populations via Annexin V - PI flow cytometry. We observed a significant increase in late apoptotic cells in Huh7 (p<0.01) and HepG2 *TREM1* KO (p<0.05) lines compared to control cells (**Figure 2F**). This finding suggests that TREM1 expression exerts an anti-apoptotic effect to support tumor cell survival, and that its silencing shifts the balance toward pro-apoptosis. To further characterize this phenomenon, we used the RT2 Apoptosis Profiler Array to screen 84 key genes involved in apoptosis and their perturbations during *TREM1* silencing. We identified significant changes in the expression of apoptosis-related genes in Huh7 *TREM1* KO cells in comparison to control (**Figure 2G**). TREM1 silencing led to significant (p<0.0001) upregulation of pro-apoptotic genes such as *BAX*, *CASP3*, and *CASP14*, indicating enhanced activation of apoptotic pathways (37, 38). Conversely, anti-apoptotic genes like *BAG1* and *BCL2*, which promote cell survival (39, 40), were significantly downregulated in *TREM1* KO cells (**Figure 2H**). A similar trend was observed in HepG2 cells, where *TREM1* silencing led to a significant (p < 0.001) upregulation of pro-apoptotic genes such as *BAX* and *CASP3*, along with downregulation of anti-apoptotic genes including *BIRC3* and *BAG1* (**Supplementary Figures 5A, B**).These findings suggest that TREM1 functions as a vital regulator of cell survival in HCC by suppressing apoptosis and promoting proliferation, migration, and cell cycle progression. TREM1 abrogation disrupts these processes, thereby impairing overall tumor cell viability.

### TREM1 promotes tumorigenicity, clonogenic potential, and spheroid formation in CD133^+^EpCAM^+^ LCSLCs

**Figure 3:**
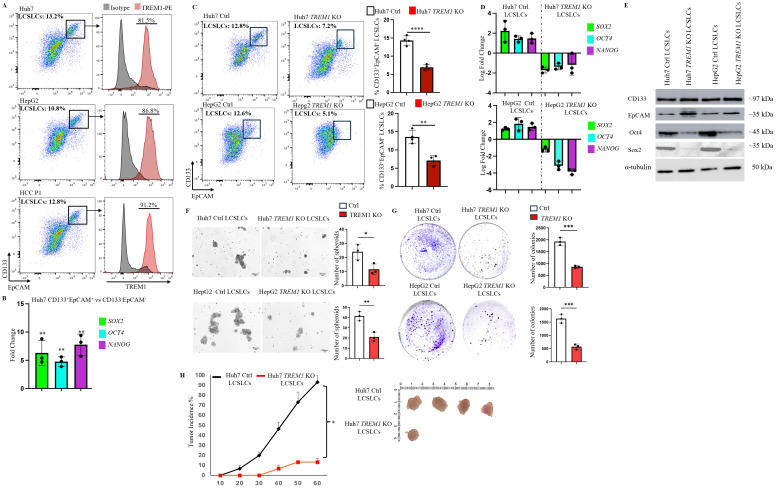
TREM1 promotes tumorigenicity, clonogenic potential and spheroid formation in CD133^+^EpCAM^+^ liver cancer stem-like cells. **(A)** Flow cytometry dot plots reveal significant TREM1 expression in CD133^+^EpCAM^+^ cells from Huh7, and HepG2 cell lines and HCC P1 patient sample. **(B)** RT-PCR analysis shows significant expression of stem cell factors in MACS-purified Huh7 CD133^+^EpCAM^+^ cells in comparison to CD133^-^EpCAM^-^ fractions (n=3 per group) **(C)** Flow cytometry dot plots depict reduction in CD133^+^Epcam^+^LCSLCs during TREM1 silencing in Huh7 and HepG2 cells (n=4 per group). **(D)** RT-PCR analysis of MACS-purified LCSLCs reveals a significant decrease in stem cell factor expression during TREM1 ablation (n=3 per group). **(E)** Western blot analysis shows significant expression of stem cell proteins in MACS purified Huh7 and HepG2 control CD133^+^EpCAM^+^ cells in comparison to *TREM1* KO CD133^+^EpCAM^+^ cells. **(F)** Spheroid formation assay shows TREM1 abrogation significantly limits spheroid formation and overall proliferation of LCSLCs. Keyence microscope was used for the acquisition of bright field images. Scale bars = 50 μm. Spheroids were counted using ImageJ (n=3 per group) **(G)** Colony formation assay demonstrates TREM1-positive LCSLCs form significantly more colonies than their KO counterparts. Representative images show *TREM1* KO and Control Huh7 and HepG2 LCSLC colonies stained using crystal violet after 14 days. Data were plotted using GraphPad Prism (n=3 per group). **(H)** Representative images of tumors from NSG CDX models. 5000 Huh7 CD133^+^EpCAM^+^ LCSLCs from Control and *TREM1* KO groups were injected subcutaneously (n=6 mice per group). The experiment was independently repeated three times for statistical analysis. ***p<0.001, ****p<0.0001.

Our data demonstrated the integral role of TREM1 in HCC, as its silencing inhibited cell proliferation and migration (**Figures 2C, D**), disrupted cell cycle progression, and enhanced apoptosis (**Figures 2E, F**). Given this profound impact, we analyzed TREM1 expression in LCSLCs, which are integral drivers of HCC tumors (41, 42). Flow cytometry analysis revealed that a significant proportion (80 ± 6%) of CD133^+^EpCAM^+^ LCSLCs in Huh7 and HepG2 cell lines and the primary HCC P1 patient sample actively expressed TREM1 (**Figure 3A**). To further validate this observation, we performed RT-PCR on FACS-sorted CD133^+^EpCAM^+^ LCSLCs from Huh7, HepG2, and HCC P1. TREM1 expression was significantly elevated in the LCSLC population compared to the corresponding CD133^-^EpCAM^-^ non-stem cell populations (**Supplementary Figure 6**). To confirm the stemness of these cells, we MACS purified Huh7 CD133^+^EpCAM^+^ and CD133^-^EpCAM^-^ cells. RT-PCR analysis showed significantly (p<0.01) higher expression of prominent stem cell factors—*SOX2*, *OCT4*, and *NANOG*—in CD133^+^EpCAM^+^ cells (43–45) compared to their negative fractions, corroborating their characterization as cancer stem-like cells (**Figure 3B**). Preliminary flow cytometry analysis revealed a significant reduction in the overall frequency of CD133^+^EpCAM^+^ LCSLCs in Huh7 (p<0.0001) and HepG2 *TREM1* KO (p<0.01) groups compared to their respective controls (**Figure 3C**). RT-PCR analysis of MACS-purified LCSLCs showed significant downregulation of *SOX2, OCT4*, and *NANOG* during TREM1 silencing in both Huh7 and HepG2 cells compared to controls (**Figure 3D**). This was further validated at the protein level using Western blot analysis (**Figure 3E**). Spheroid formation assays using purified LCSLCs demonstrated that TREM1 silencing significantly inhibited spheroid formation capacity in both Huh7 (p<0.05) and HepG2 cells (p<0.01) (**Figure 3F**). Additionally, colony formation assays revealed that TREM1 ablation significantly inhibited clonogenicity and self-renewal capacity of LCSLCs in both cell lines (**Figure 3G**). We also evaluated the tumorigenic potential of TREM1-deficient and TREM1-sufficient Huh7 LCSLCs in an *in vivo* setting. We implanted 5000 FACS-purified Huh7 CD133^+^EpCAM^+^ LCSLCs from *TREM1* KO or control groups into immunodeficient NSG mice to establish a CDX model (**Figure 3H**). Over a 60-day period, only 1 out of 6 mice implanted with *TREM1* KO LCSLCs developed tumors, whereas in the TREM1-positive implants, tumor formation was significantly higher, with 4 out of 6 developing tumors. This stark contrast in tumor initiation underscores the crucial role of TREM1 in driving the tumorigenicity of LCSLCs.

### Bulk RNA-seq reveals the impact of TREM1 silencing on cancer stemness and distinct signaling pathways in CD133^+^EpCAM^+^ Huh7 and HepG2 cells

**Figure 4:**
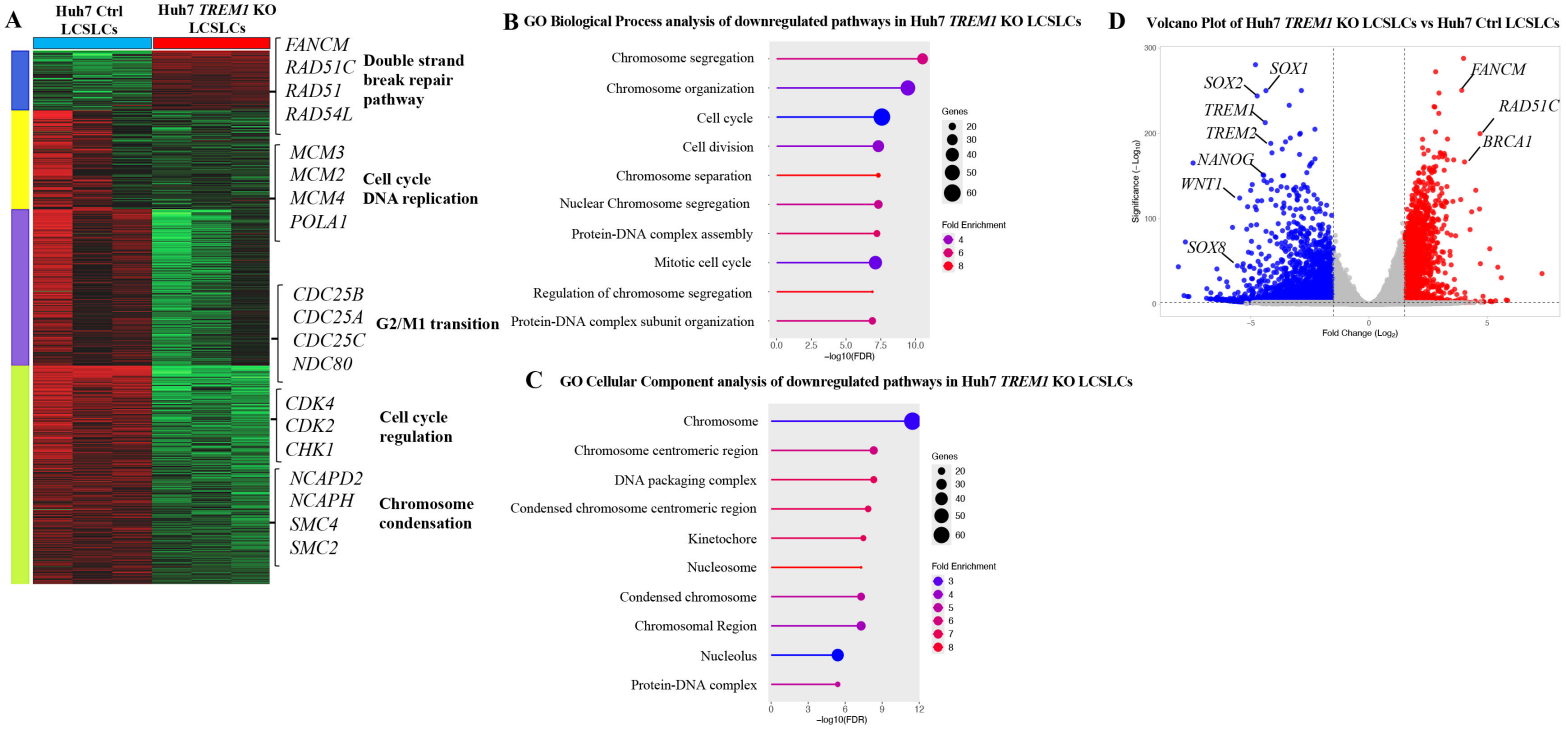
Bulk RNA sequencing and pathway analysis of CD133^+^EpCAM^+^ cells from Huh7 control and *TREM1* KO cell lines. **(A)** Heat map created using K-means clustering of the top 10,000 differentially expressed genes (DEGs) reveals that *TREM1* KO in Huh7 LCSLCs significantly impacts cell proliferation pathways. Each sample was analyzed in triplicate. **(B)** Pathway analysis using Gene Set Enrichment Analysis (GSEA). The lollipop plot highlights the downregulated Gene Ontology (GO) Biological Process pathways in *TREM1* KO Huh7 LCSLCs. **(C)** The lollipop plot shows the GO cellular components inhibited by the *TREM1* KO, emphasizing the nuclear structures and complexes impacted. **(D)** Volcano plot displays the DEGs between Huh7 Ctrl and Huh7 *TREM1* KO CD133^+^EpCAM^+^ cells. Genes with a log2 fold change > 1 and adjusted *p-*value < 0.05 are highlighted in red, indicating significant upregulation, while those with a log2 fold change < -1 and adjusted *p*-value < 0.05 are highlighted in blue, indicating significant downregulation. This plot underscores the downregulation of TREM1 and cancer stem cell-associated genes in the Huh7 CD133^+^EpCAM^+^ *TREM1* KO cells.

**Figure 5:**
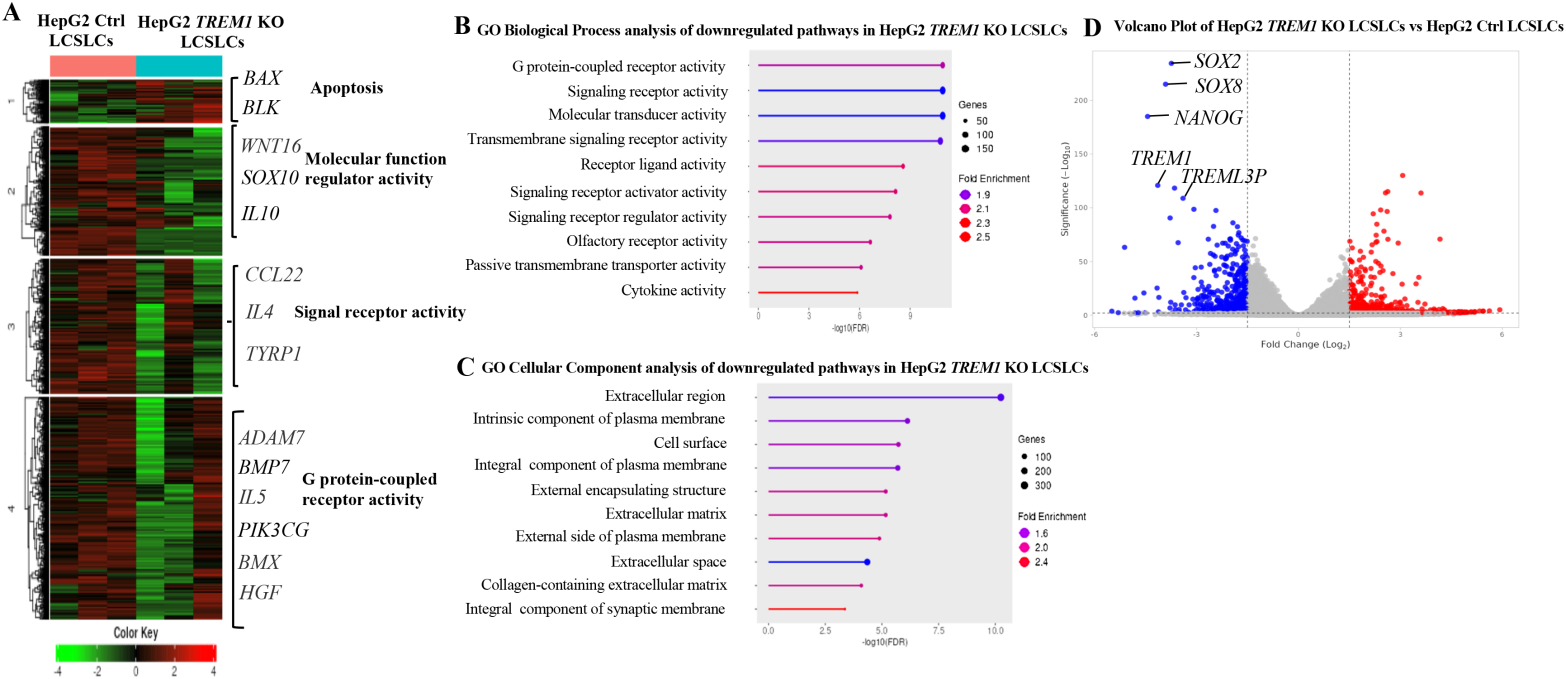
Bulk RNA sequencing and pathway analysis of CD133^+^EpCAM^+^ cells from HepG2 control and *TREM1* KO cell lines. **(A)** Heat map created using K-means clustering of the top 10,000 differentially expressed genes (DEGs) reveals that *TREM1* KO in HepG2 LCSLCs predominantly affects extracellular pathways. Each sample was analyzed in triplicate. **(B)** Pathway analysis using GSEA. The lollipop plot highlights the downregulated pathways in *TREM1* KO HepG2 LCSLCs, focusing on significant extracellular pathways affected by the knockout. **(C)** The lollipop plot shows the GO cellular components inhibited by the *TREM1* KO, emphasizing the extracellular structures and complexes impacted. **(D)** Volcano plot displays the DEGs between HepG2 Control and HepG2 KO CD133^+^EpCAM^+^ cells. Genes with a log2 fold change > 1 and adjusted *p*-value < 0.05 are highlighted in red, indicating significant upregulation, while those with a log2 fold change < -1 and adjusted *p*-value < 0.05 are highlighted in blue, indicating significant downregulation. This plot highlights the downregulation of *TREM1* and cancer stem cell-associated genes in the HepG2 CD133^+^EpCAM^+^ *TREM1* KO cells.

Our previous data demonstrated the integral role of TREM1 in maintaining the tumorigenicity of CD133^+^EpCAM^+^ LCSLCs in Huh7 and HepG2 cells. To determine the molecular function of TREM1, we performed bulk RNA sequencing of FACS-purified CD133^+^EpCAM^+^ LCSLCs from Huh7 and HepG2, *TREM1* KO, and control tumors. K-means clustering of the top 10,000 differentially expressed genes between TREM1-control and *TREM1* KO cells resolved four different clusters. In Huh7 LCSLCs, TREM1 silencing led to the downregulation of genes involved in cell cycle regulation (*CDK4, CDK2, CHK1*) (46), DNA replication (*MCM3, MCM2, MCM4, POLA1*) (47, 48), G2/M transition (*CDC25B, CDC25A, CDC25C, NDC80*) (49, 50), and chromosome condensation (*NCAPD2, NCAPH, SMC4, SMC2*) (51), while genes associated with the double-strand break repair pathway *(FANCM, RAD51C, RAD51, RAD54L*) (52, 53) were upregulated (**Figure 4A**). Gene Set Enrichment Analysis (GSEA) incorporating the Gene Ontology (GO) biological process pathway identified significant downregulation in pathways related to chromosome separation, organization, and protein-DNA complex assembly (**Figure 4B**). GO cellular component analysis showed that a significant number of genes downregulated during TREM1 silencing were localized in the chromosome, nucleus, and nucleosome regions (**Figure 4C**). These observations indicate that in Huh7 LCSLCs, TREM1 silencing primarily affects nuclear structures, cell division processes, and chromosome complexes. Volcano plots revealed that TREM1 genetic ablation led to a significant decrease in stemness-related genes such as *SOX1, SOX2, NANOG, WNT1*, and *SOX8* (54) in Huh7 LCSLCs (**Figure 4D**). Conversely, in HepG2 LCSLCs, genes involved in molecular function regulation (*WNT16, SOX10, IL10*) (55), signal receptor activity (*CCL22, IL4, TYRP1*), and G protein-coupled receptor (GPCR) activity (*ADAM7, BMP7, IL5, PIK3CG, BMX, HGF*) (56) were downregulated, whereas pro-apoptotic genes (*BAX, BLK*) were upregulated during TREM1 ablation (**Figure 5A**). GO biological process pathway enrichment analysis revealed a significant reduction in cytokine activity, receptor-ligand interactions, and signaling receptor activity, suggesting that TREM1 silencing in HepG2 LCSLCs predominantly affects extracellular signaling pathways (**Figures 5B, C**). Volcano plots revealed that along with TREM1, several cancer stemness-related genes including *NANOG, SOX2, SOX8*, and *WNT16* (57–59) were significantly downregulated (**Figure 5D**). These findings suggest that TREM1 silencing alters key signaling pathways in a cell-type-specific manner, disrupting nuclear functions in Huh7 cells and extracellular signaling in HepG2 cells, while also impairing cancer stem cell properties in both cell lines.

### TREM1 inhibition via VJDT depletes LCSLCs, reduces tumor size, and decreases spheroid formation

**Figure 6:**
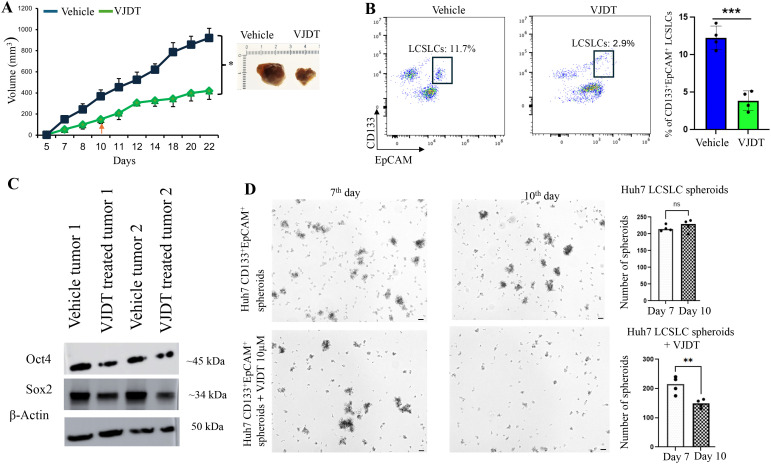
TREM1 inhibition via VJDT depletes LCSLCs, reduces tumor size, and decreases spheroid formation. **(A)** Tumor growth curves for Huh7 vehicle and VJDT treated mice (mean ± SEM, n=5 mice/group). Representative images of tumors from indicated groups on day 22. **(B)** Flow cytometry analysis shows a significant reduction in CD133^+^EpCAM^+^ LCSLCs in VJDT-treated tumors compared to the vehicle (n=4 per group). **(C)** Western blot analysis of two vehicle-treated and two VJDT-treated tumors shows reduced expression of stem cell-related proteins in VJDT-treated tumors. **(D)** Representative images from the spheroid formation assay demonstrate reduced spheroid formation following VJDT treatment. Scale bar = 50 µm. Spheroids were counted using ImageJ. **p<0.01, ***p<0.001, ns-not significant.

We previously demonstrated that *TREM1* KO LCSLCs exhibit significantly reduced tumorigenic and spheroid formation capabilities. To further validate these findings, we utilized VJDT, a small molecular inhibitor of TREM1 (7, 9, 60, 61). We subcutaneously injected 1x10^6^ Huh7 cells into 10 NSG mice. On day 10, five of these mice were treated with 20 mg/kg VJDT every alternative day until day 22. VJDT treatment significantly (p<0.05) reduced overall tumor volumes in comparison to the vehicle group (**Figure 6A**). Tumors were digested into single-cell suspensions for flow cytometry analysis, which revealed a significant reduction in the percentage of CD133^+^EpCAM^+^ LCSLCs in the VJDT-treated tumors (**Figure 6B**). Western blot analysis of Huh7 LCSLC tumors revealed decreased expression of stem cell-related proteins such as Sox2, Nanog, and Oct4 in the VJDT-treated group (**Figure 6C**). Additionally, we isolated 5000 Huh7 and HepG2 CD133^+^EpCAM^+^ LCSLCs using MACS and performed spheroid formation assays. Spheroids were formed by day 5. The spheroids were treated with 10 µM VJDT or vehicle from day 7. VJDT treatment significantly (p<0.01) inhibited spheroid formation by day 10 in Huh7 (**Figure 6D**) and HepG2 (**Supplementary Figure 7A**). Furthermore, RT PCR analysis of VJDT treated spheroids exhibited significant downregulation in key genes involved in molecular function (*SOX10, IL10*), signal receptor activity (*CCL22, IL4*) and GPCR activity (*ADAM7, IL5*) (**Supplementary Figure 7B**), corroborating previous results shown in bulk RNA sequencing. These findings confirm that TREM1 inhibition via VJDT effectively depletes the number of LCSLCs, reduces tumor size, and decreases spheroid formation. This also validates the results we obtained from using Huh7 CRISPR-Cas9 *TREM1* KO cells and highlights the potential of VJDT as a therapeutic agent in targeting liver cancer stem-like cells.

## Discussion

TREM1 is a significant proinflammatory molecule in the TME of solid tumors such as lung, gastric, and HCC. TREM1 is primarily expressed in myeloid cells (monocytes, macrophages, neutrophils) and possibly in malignant cells, although its exact role in tumor promotion is not yet well understood (7). Herein numerous studies have investigated the direct role of TREM1 in cancer cells, proving consistent observation of TREM1 protein expression in tumors and their cancer promoting role (62). In hematological malignancies TREM1 expression is elevated in hematopoietic stem cells during oncogenic stress or persistent DNA damage (63). In thyroid and prostate cancer cells TREM1 expression is regulated by epigenetic modifications, specifically hypomethylation of CpG site Cδ06196379 in the TREM1 promoter region and has significant prognostic value in thyroid tumors (64). In HCC prior publications have demonstrated that over expression of TREM1 can significantly enhance tumor cell proliferation and survival. This involved TREM1 induced upregulation of IL-6, STAT3, ERK1/2 and AKT pathways (65). We have previously reported that TREM1 is upregulated in and is responsible for activation of Kupffer cells and hepatic stellate cells in HCC development and progression (10). Our current study provides compelling evidence that TREM1 plays a crucial role in the tumorigenicity and maintenance of LCSLCs in HCC. Analysis of the TCGA database revealed a consistent overexpression of TREM1 in aggressive LIHC subtypes and its correlation with poor prognosis highlights its importance in HCC progression (66). Furthermore, correlation analysis of the LIHC cohorts revealed that TREM1 expression is associated with numerous cell cycle specific genes and cell regulation, separate from its association with inflammatory pathways. This was an interesting phenomenon as it implies a role for TREM1 separate from its immunomodulatory nature in the TME. Furthermore, TREM1 ablation utilizing CRISPR-Cas9 revealed a marked decrease in cell proliferation and survival in both HepG2 and Huh7 cell lines. Interestingly TREM1 ablation led to significant reduction of CD133^+^EpCAM^+^ LCSLCs in both cell lines. Additional *in vitro* assays demonstrated that TREM1 silencing significantly reduced the clonogenicity and proliferation capacity of LCSLCs. Finally, cell line xenograft studies using FACS purified LCSLCs revealed that TREM1 silencing significantly abrogated their tumorigenic capacity. To determine the molecular function of TREM1 in LCSLCs we performed bulk RNA sequencing of FACS purified LCSLCs. GSEA of our transcriptomic data revealed that TREM1 silencing disrupts key signaling pathways in a cell-type-specific manner. In Huh7 LCSLCs, TREM1 ablation primarily affected nuclear functions and cell division processes, while in HepG2 cells, it predominantly impacted extracellular signaling pathways. This differential impact underscores the complexity of TREM1’s role in HCC and suggests that targeted therapies may need to be tailored to specific cellular contexts.

The downregulation of stemness-related genes such as *SOX2, NANOG*, and *OCT4* during TREM1 silencing supports the role of TREM1 in maintaining the stem-like properties of LCSLCs. This reduction in LCSLC populations upon TREM1 inhibition suggests that targeting TREM1 could reduce the likelihood of tumor recurrence. These observations are consistent with previous publications demonstrating the integral role of TREM1 in leukemia stem cells. Importantly TREM1 ablation in preleukemic stem cells compromised proliferation and delayed leukemia *in vivo* (63). Our observations signify a similar role of TREM1 in LCSLCs of HCC. By employing both CRISPR-Cas9-mediated KO and the small molecule inhibitor VJDT, we demonstrated that TREM1 inhibition significantly reduces tumor size, depletes the number of LCSLCs, and impairs spheroid formation. These findings open new avenues for therapeutic interventions aimed at eradicating cancer stem cells and improving patient outcomes. Furthermore, these findings underscore the potential of TREM1 as a therapeutic target in HCC and suggests its direct role in the cells independent of its effects in the TME (67). This is exceptionally fortuitous as our prior studies demonstrated that TREM1 inhibition remodels the TME to a more immunopermissive state and augments anti-PD-1 treatment to overcome its resistance in melanoma (9). In combination with our current observations, inhibiting TREM1 can target its intrinsic expression in cancer cells and its extrinsic role in the TME.

While our study provides valuable insights, it is not without limitations. The use of established cell lines and xenograft models, though informative, may not fully capture the complexity of human HCC. To bridge this gap, we included a primary HCC patient tumor sample to examine TREM1 expression in CD133^+^EpCAM^+^ LCSLCs. Although the inclusion of a single patient sample does not permit statistical inference, its consistent expression pattern provided supportive evidence in line with our *in vitro* findings in Huh7 and HepG2 cell lines, which form the basis of our primary conclusions. Future studies should aim to validate these observations across larger patient cohorts and explore the therapeutic potential of TREM1 inhibitors in combination with current treatment modalities. Further research is also warranted to elucidate the molecular mechanisms by which TREM1 regulates cancer stemness in HCC. Finally, examining TREM1’s role across additional tumor types may broaden its utility as a cancer stem cell–directed therapeutic target.

Our study identifies TREM1 as a critical regulator of liver cancer stem-like cells (LCSLCs) in hepatocellular carcinoma (HCC), exhibiting distinct tumor-intrinsic functions beyond its well-established immunomodulatory roles in the tumor microenvironment. Through both genetic and pharmacological approaches, we demonstrate that TREM1 supports LCSLC proliferation, self-renewal, and tumor-initiating capacity via context-specific transcriptional programs. These findings not only expand our understanding of TREM1 biology but also establish it as a compelling dual-action therapeutic target. By targeting TREM1, it may be possible to simultaneously impair LCSLC-driven tumor progression and enhance antitumor immunity. This work supports the development of novel therapeutic strategies aimed at improving prognosis and treatment outcomes in HCC by reducing LCSLC stemness, and thereby limiting tumor recurrence, metastasis, and resistance to therapy.

## Data Availability

The data generated or analyzed during this study are included in this manuscript and its **Supplementary Material**. The datasets presented in this study can be found in online repositories. The names of the repository/repositories and accession number(s) can be found below: ArrayExpress database at EMBL-EBI (www.ebi.ac.uk/arrayexpress) under accession number E-MTAB-15204.
