# Supplementary material for: TREM1 is essential for maintaining stemness of liver cancer stem-like cells in hepatocellular carcinoma
Source: Front Immunol. 2025 Jul 3;16:1618342. doi: 10.3389/fimmu.2025.1618342 (PMC12267286; doi:10.3389/fimmu.2025.1618342)
Supplement: Supplementary file 1 [file DataSheet1.docx]

Supplementary Material

Supplementary Figure 1……………………………………………………………………………………….….2

Supplementary Figure 2……………………………………………………………………………………….….3

Supplementary Figure 3……………………………………………………………………………………….….4

Supplementary Figure 4…………………………………………………………………………………………..5

Supplementary Figure 5…………………………………………………………………………………………..6

Supplementary Figure 6…………………………………………………………………………………………..7

Supplementary Figure 7…………………………………………………………………………………………..8

Supplementary Table……………………………………………………………………………………………...9

**A** Design and development of CRISPR-Cas9 HepG2 *TREM1* KO cell lines

Supplementary Figure 1: Design and development of CRISPR-Cas9 HepG2 *TREM1 KO* cell lines. (A) Schematic representation of guide RNA sequence and its target within Exon 2 of *TREM1*. (B) PCR primers used to validate positive clones with the deletion of 50bp within Exon 2. (C) Sanger’s sequencing validation of deletion in the depicted clones.

**Supplementary Figure 1**

**Supplementary Figure 2**

Supplementary Figure 2: Design and development of CRISPR-Cas9 Huh7 *TREM1 KO* cell lines. (A) Schematic representation of guide RNA sequence and its target within Exon 2 of *TREM1*. (B) PCR primers used to validate positive clones with the deletion of 169bp within Exon 2. (C) Sanger’s sequencing validation of deletion in the depicted clones.

**A** Design and development of CRISPR-Cas9 Huh7 *TREM1* KO cell lines

**Supplementary Figure 3**


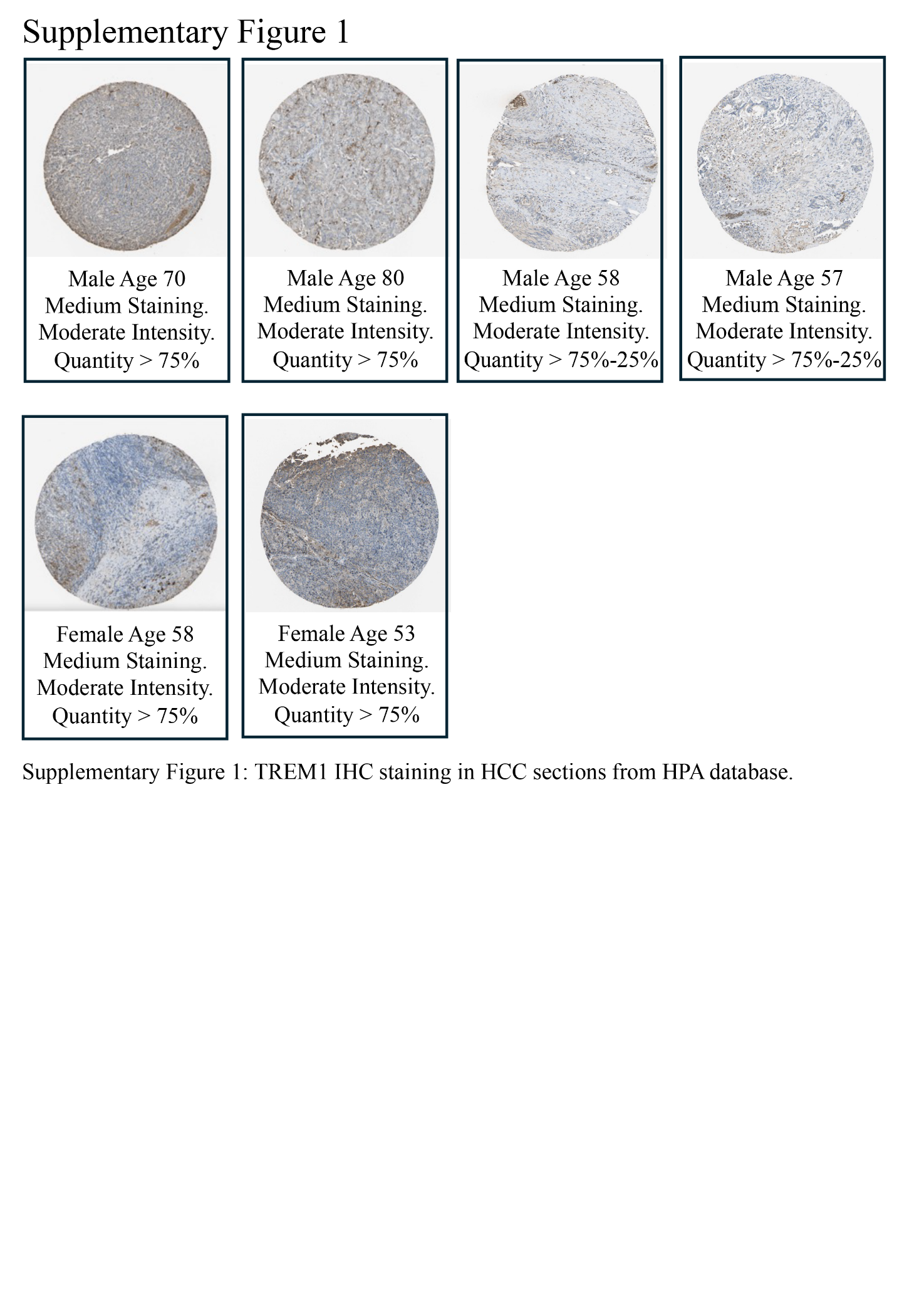


Supplementary Figure 3: TREM1 IHC staining in HCC sections from HPA database.

**Supplementary Figure 4**

**B**

**A**

TREM1

TREM1

Supplementary Figure 4: Flow cytometry analysis of TREM1 expression. (A) Flow cytometry histogram plots depict TREM1 expression in Huh7 Control (Blue Line) and Huh7 *TREM1* KO (Red Line) cell lines. Filled histogram represents isotype control. (B) Flow cytometry histogram plots depict TREM1 expression in HepG2 Control (Blue Line) and HepG2 *TREM1* KO (Red Line) cell lines. Filled histogram represents isotype control.

**Supplementary Figure 5**

HepG2 *TREM1* KO

HepG2 Control

HepG2 Control

HepG2 Control

**B**

**A**

Supplementary Figure 5: RT PCR analysis of human apoptotic gene expression. (A) RT2 profiler array used to compile heatmap depicting the expression of 84 key genes associated with apoptosis. Upregulated genes (red) and downregulated genes (blue) in HepG2 *TREM1* KO cells in comparison to HepG2 Control. (B) Upregulation of key pro-apoptotic genes and downregulation of specific anti-apoptotic genes in HepG2 *TREM1* KO cells compared to the control group.

RT-PCR analysis of MACS-purified CD133⁺EpCAM⁺ LCSLCs shows elevated TREM1 expression compared to non-LCSLC fractions in the indicated groups.

**Supplementary Figure 6**

Supplementary Figure 6: TREM1 expression in CD133⁺EpCAM⁺ liver cancer stem-like cells (LCSLCs).RT-PCR analysis of FACS-purified CD133⁺EpCAM⁺ LCSLCs shows elevated *TREM1* expression compared to non-LCSLC fractions in the indicated groups. In each group *TREM1* expression of CD133^-^EpCAM^-^ cells were taken as the baseline for comparative fold change.

**Supplementary Figure 7**

**A B**


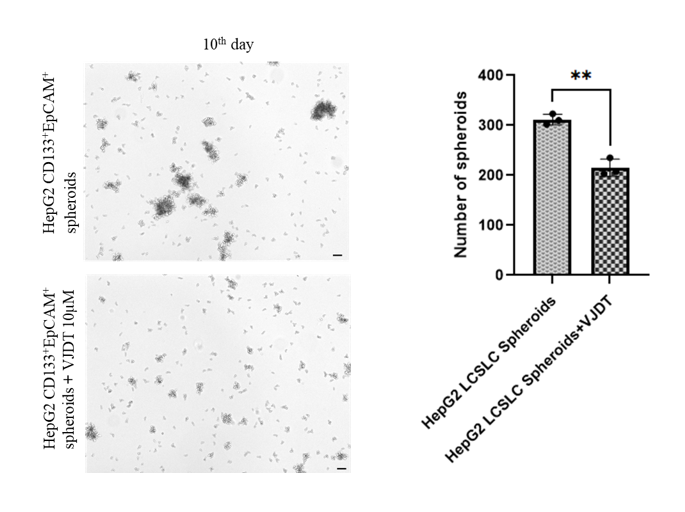


*SOX10*

*IL10*

*CCL22*

*IL4*

*ADAM7*

*IL5*

*HepG2 LCSLCs Control*

*HepG2 LCSLCs VJDT*

Log Fold Change

Supplementary Figure 7: VJDT treatment of HepG2 LCSLCs (A) Spheroid formation assay demonstrating reduced spheroid formation in HepG2 LCSLCs following VJDT treatment. Scale bar = 50 µm. Spheroids were counted using ImageJ. (B) RT PCR analysis of HepG2 LCSLCs spheroids with TREM1 inhibition by VJDT. Downregulation of key genes in MACS-purified CD133⁺EpCAM⁺ LCSLCs after VJDT treatment. Control group used as baseline for comparative Fold change analysis. (n=3 per group).

| Gene | Sequences, 5’-3’ | |
| --- | --- | --- |
|  | Forward | Reverse |
| *TREM1* | CGATGTCTCCACTCCTGACTCT | CAGCAAACAGGACAGAGAAGACC |
| *SOX2* | GCTACAGCATGATGCAGGACCA | TCTGCGAGCTGGTCATGGAGTT |
| *NANOG* | CTCCAACATCCTGAACCTCAGC | CGTCACACCATTGCTATTCTTCG |
| *OCT4* | CCTGAAGCAGAAGAGGATCACC | AAAGCGGCAGATGGTCGTTTGG |
| *GAPDH* | GTCTCCTCTGACTTCAACAGCG | ACCACCCTGTTGCTGTAGCCAA |
| *SOX10* | ATGAACGCCTTCATGGTGTGGG | CGCTTGTCACTTTCGTTCAGCAG |
| *IL10* | TCTCCGAGATGCCTTCAGCAGA | TCAGACAAGGCTTGGCAACCCA |
| *IL4* | CCGTAACAGACATCTTTGCTGCC | GAGTGTCCTTCTCATGGTGGCT |
| *CCL22* | TCCTGGGTTCAAGCGATTCTCC | GTCAGGAGTTCAAGACCAGCCT |
| *TYRP1* | TCTCAATGGCGAGTGGTCTGTG | CCTGTGGTTCAGGAAGACGTTG |
| *ADAM7* | GCCAGGATTTACTTGTGCAGAAG | CCTGAACTGGTCCTTAGGACAG |
| *BMP7* | GAGTGTGCCTTCCCTCTGAACT | AGGACGGAGATGGCATTGAGCT |
| *IL5* | GGAATAGGCACACTGGAGAGTC | CTCTCCGTCTTTCTTCTCCACAC |
| *BAX* | TCAGGATGCGTCCACCAAGAAG | TGTGTCCACGGCGGCAATCATC |
| *CASP3* | GGAAGCGAATCAATGGACTCTGG | GCATCGACATCTGTACCAGACC |
| *CASP14* | GGTGGATGTGTTCACGAAGAGG | CCTTCTTGAACCAGCTCTGCTTC |
| *BAG1* | GTGAACCAGTTGTCCAAGACCTG | CAAGTGCTGACAACGGTGTTTCC |
| *BCL2* | ATCGCCCTGTGGATGACTGAGT | GCCAGGAGAAATCAAACAGAGGC |

**Supplementary Table**
