# Supplementary figures and images for: TREM1 is essential for maintaining stemness of liver cancer stem-like cells in hepatocellular carcinoma
Source: Front Immunol. 2025 Jul 3;16:1618342. doi: 10.3389/fimmu.2025.1618342 (PMC12267286; doi:10.3389/fimmu.2025.1618342)

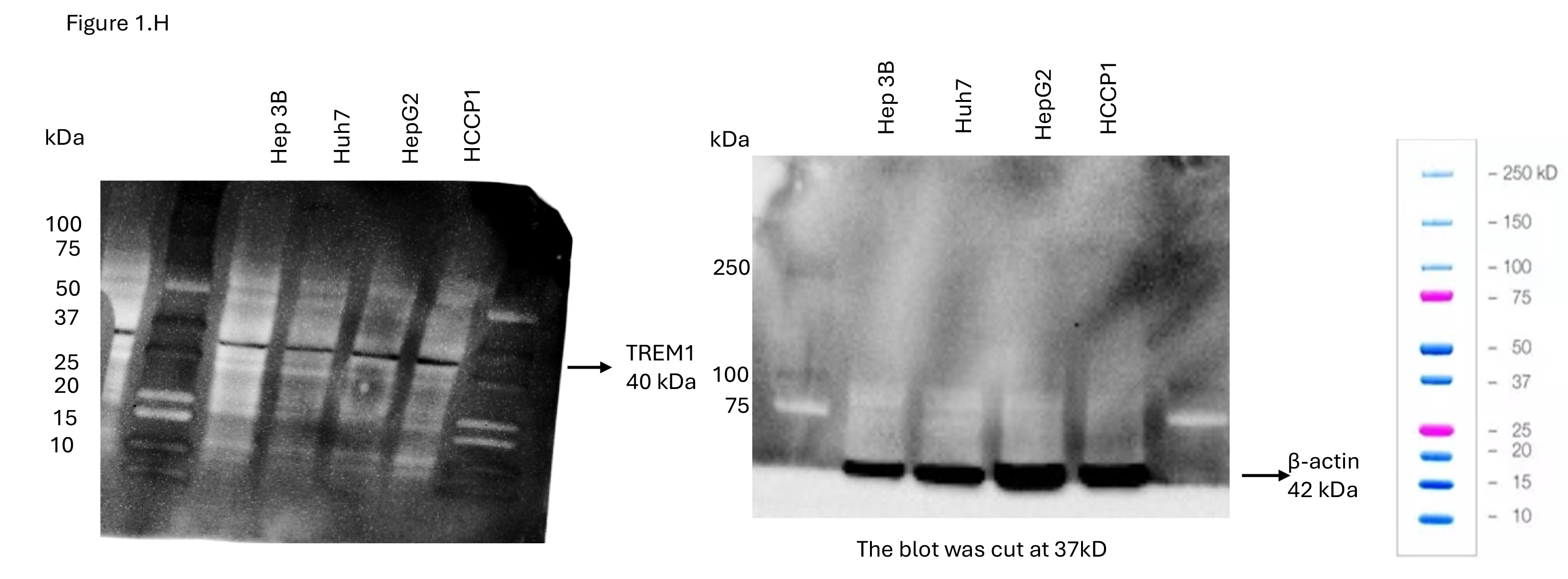

Supplement: Supplementary file 2 [file Image1.tif]

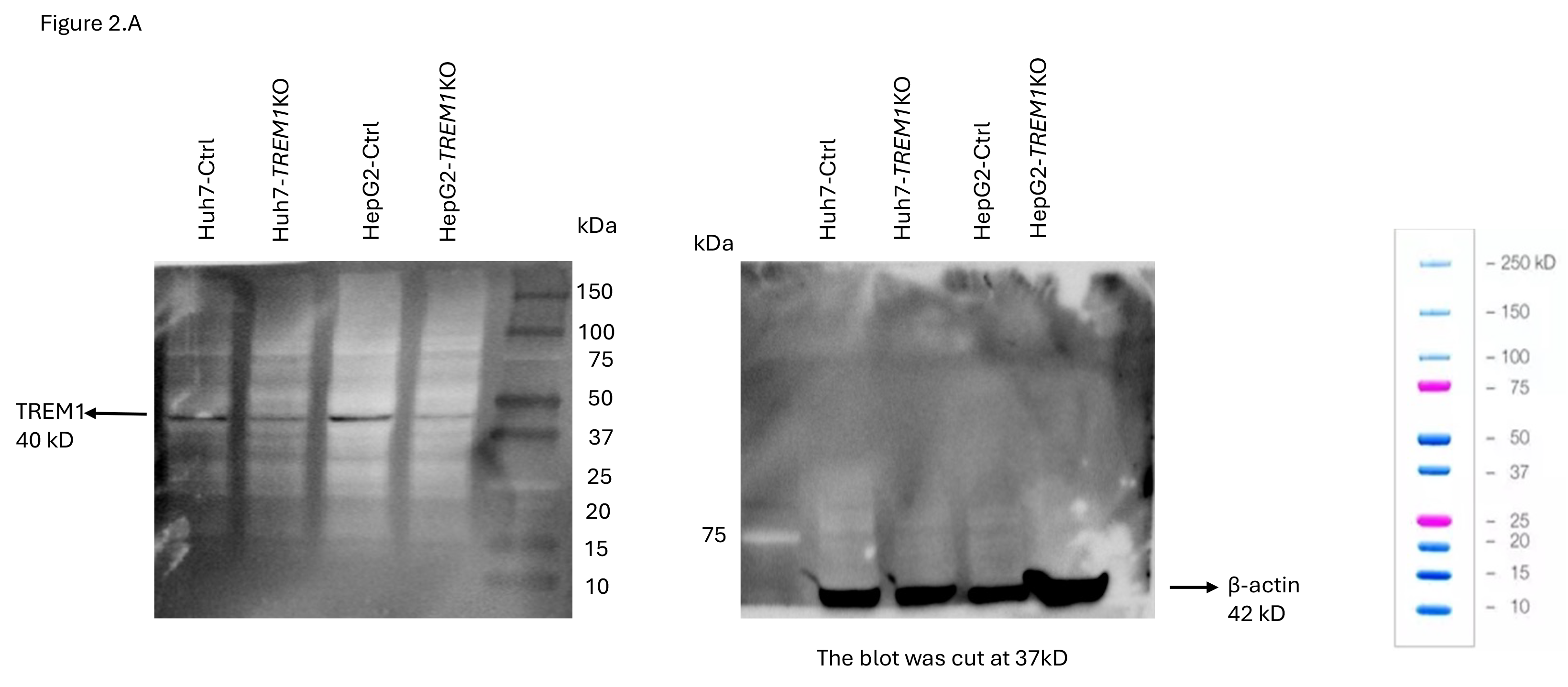

Supplement: Supplementary file 3 [file Image2.tif]

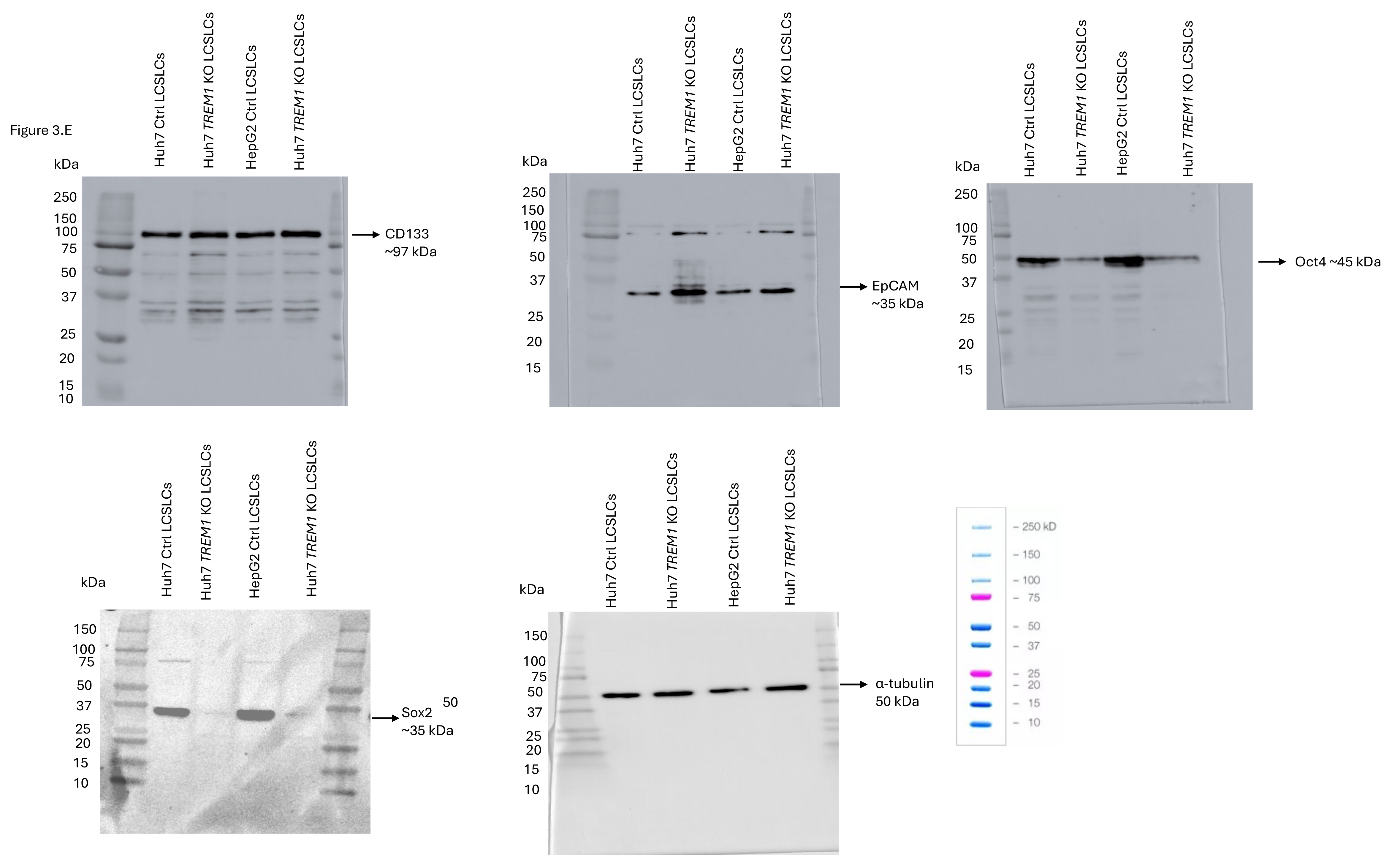

Supplement: Supplementary file 4 [file Image3.tif]

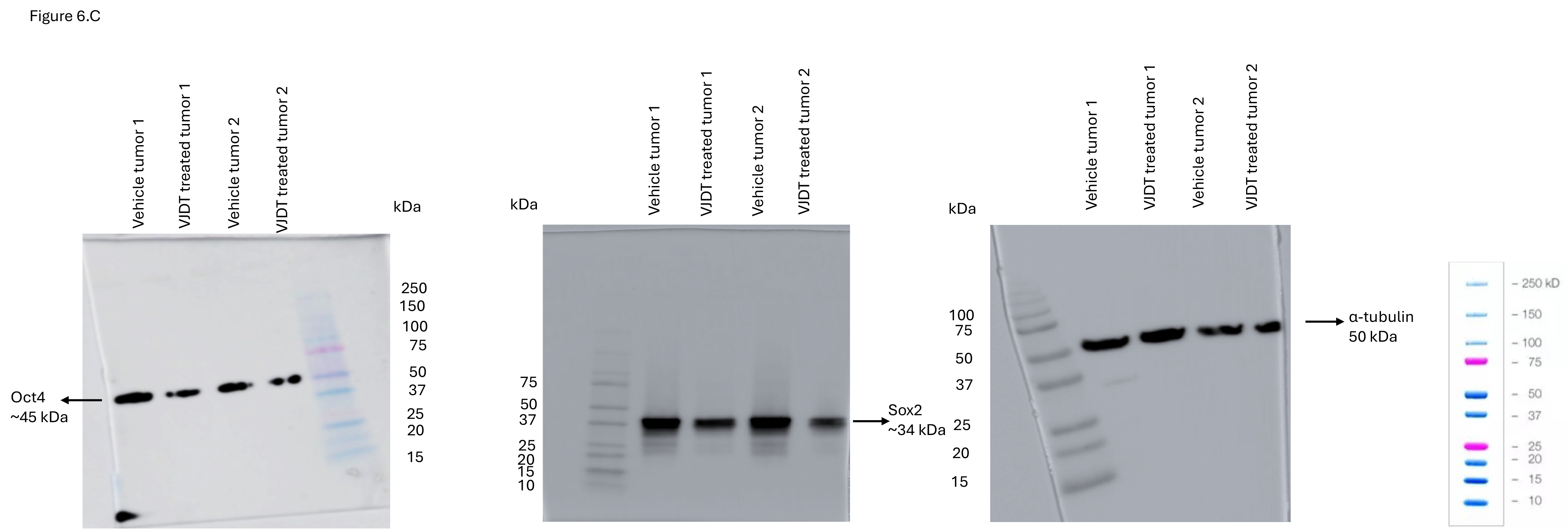

Supplement: Supplementary file 5 [file Image4.tif]
